# Intensive care subspecialty in turkey: what do emergency medicine physicians think?

**DOI:** 10.1016/j.clinsp.2026.101092

**Published:** 2026-09-05

**Authors:** Esat Barut, Ramazan Giden, Kemal Barut, İbrahim Halil Yasak, Songül Araç

**Affiliations:** a Emergency Department, Harran University, Şanlıurfa, Turkey; b Emergency Department, Gazi Yasargil Training and Research Hospital, Diyarbakır, Turkey

**Keywords:** Emergency medicine, Intensive care, Subspecialty, Training, Physician shortage

## Abstract

**Objective:**

The aim of this study is to investigate the tendencies, self-perceived knowledge, perceived barriers, and expectations of Emergency Medicine (EM) physicians working in Turkey regarding the Intensive Care (IC) subspecialty.

**Methods:**

A cross-sectional, descriptive survey study was conducted between July and September 2025. A total of 304 EM physicians actively working in Turkey were included in the study. The survey form consisted of 12 questions covering demographic information, self-perceived knowledge, experience, tendency, motivation, opinions, and expectations. Data were analyzed using SPSS 28.0 with chi-square tests for categorical variables and multivariate logistic regression for factors associated with subspecialty tendency and self-perceived knowledge.

**Results:**

Among participants, 80.6% had experience in patient follow-up in IC. While 43.1% were considering pursuing an IC subspecialty, only 9.5% had detailed information about it. Duration of practice, gender, self-perceived knowledge, and IC experience were significantly associated with subspecialty tendency (p < 0.001 for all). Multivariate analysis identified self-perceived knowledge (OR = 3.07) and IC experience (OR = 6.70) as independently associated factors for subspecialty tendency, while IC experience was the only significant associated factor for self-perceived knowledge (p < 0.001 for all). Primary motivations included better working conditions and salary, whereas barriers were uncertainty and compulsory service. Top suggestions included strengthening communication and cooperation, increasing self-perceived knowledge, and regulating compulsory service.

**Conclusion:**

EM physicians show notable interest in IC subspecialty, with self-perceived knowledge and IC experience as the strongest predictors of preference. Strategies to increase subspecialty tendency should include implementing informational programs, enhancing IC rotation quality, improving working conditions, and reviewing compulsory service regulations.

## Highlights

• 43.1% of EM specialists in Turkey are considering the IC subspecialty.
• Knowledge level and IC experience strongly predict subspecialty preference.
• Better working conditions and salary are the primary motivations.
• Uncertainty and compulsory service are the main barriers.
• Informational programs and improved rotations are needed.

## Introduction

In Turkey, IC services were initiated in 1959 with the establishment of the first reanimation service at Istanbul University Hospital.^1^ However, the recognition of IC medicine as a specialty and its attainment of subspecialty status occurred in the early 2000s. In 2002, with the Regulation on Specialization in Medicine, the IC subspecialty in Turkey was accepted as a subspecialty field established upon six main disciplines (anesthesiology, internal medicine, pulmonary diseases, neurology, pediatrics, and general surgery).^2^ Emergency Medicine (EM) physicians gained the right to participate in the IC subspecialty after many years of effort. On March 1, 2024, it was approved by the Turkish Grand National Assembly, and the “Intensive Care” subspecialty, the first for EM, was accepted.^3^

Emergency departments, as units where critical patients are first evaluated and stabilized, are areas where vital interventions are performed before transfer to IC units.^4^ The training and practice of EM physicians in the field of IC is a common practice worldwide, especially in the USA. In the United States, certification in the IC subspecialty has been made possible for EM physicians since 2011.^5^

In Turkey, IC services are gaining increasing importance due to the growing patient load and aging population. The COVID-19 pandemic, in particular, has highlighted the critical importance of IC capacity and the number of physicians in Turkey.^6^ This situation has revealed the need to increase the number of main disciplines that can access the IC subspecialty and to train more physicians in this field. The participation of EM physicians in the IC subspecialty represents a considerable potential in meeting this need. The experience of EM physicians in critical patient management, their mastery of resuscitation skills, and their ability to cope with emergencies are qualities that can provide valuable contributions to the field of IC.

In the national and international literature, various studies are available regarding the IC subspecialty training of EM physicians and their experiences in this field.^7^, ^8^, ^9^, ^10^, ^11^ The main purpose of our study is to investigate the tendencies, self-perceived knowledge, perceived barriers, and expectations of EM physicians working in Turkey regarding the IC subspecialty.

## Materials and methods

This study was designed as a cross-sectional, descriptive survey study to investigate the opinions of EM physicians working in Turkey regarding the IC subspecialty and was reported in accordance with the STROBE (Strengthening the Reporting of Observational Studies in Epidemiology) guidelines. The study was conducted between July and September 2025.

According to the data from the Emergency Medicine Association of Turkey (EMAT) (November 2021), there are approximately 2492 EM physicians in our country.^12^ Projecting a consistent growth rate based on the annual number of graduating residents, the estimated target population for 2025 was conservatively approximated at 4000 to 4500 physicians. Based on this estimate, the minimum required sample size was calculated as 252 participants for a 95% confidence level and a ± 6% margin of error.

Participants were recruited using convenience sampling. This distribution strategy made it infeasible to determine the exact number of physicians who received the survey invitation; thus a precise response rate could not be calculated. A total of 326 physicians responded to the survey. After applying exclusion criteria (14 residents, 5 retired physicians, and 3 incomplete responses), 304 responses were included in the final analysis, exceeding the required sample size.

Inclusion criteria: EM physicians actively working in Turkey who voluntarily completed the survey. Exclusion criteria: EM residents, retired physicians, and incomplete responses.

The survey form was developed based on a comprehensive literature review. To ensure content validity, clarity, and appropriateness, the draft survey was reviewed by five expert physicians. Following their feedback, the revised survey underwent a pilot test with 15 emergency physicians to assess its comprehensibility and identify any potential ambiguities prior to final distribution.

The survey consisted of 12 questions covering demographics (2), self-perceived knowledge and experience (2), tendency and motivation (3), opinions and expectations (3), and open-ended questions (2).

The data collection process was carried out online using the Google Forms platform. The survey link was delivered to EM physicians through associations, groups, and social communication networks. To prevent duplicate responses, the survey was configured to limit responses to one per Google account. The data collection process continued for 3-months, and participants were informed that the survey was based on voluntariness and that their data would be used only for scientific purposes.

Data analysis was performed using the SPSS 28.0 (Statistical Package for Social Sciences) program. For descriptive statistics, number, percentage, mean, and standard deviation values were used. The relationships between categorical variables were evaluated with the Chi-Square (χ²) test.

In multiple-response questions, participants were allowed to mark more than one option, and each option was coded as a separate variable, and frequency analyses were performed. In conditional questions, participants answered only the questions appropriate to their situation. A qualitative thematic analysis was conducted for the two optional open-ended questions to identify patterns within the data. An inductive coding approach was utilized, allowing themes to emerge directly from the participants' responses without pre-existing categories. Two independent researchers coded the responses and developed a coding framework. Inter-rater reliability was assessed using Cohen’s kappa coefficient to ensure consistency between the coders. Discrepancies were resolved through discussion until a consensus was reached. The open-ended questions were optional, and those who did not respond were excluded from the analysis.

A multivariate logistic regression analysis was conducted to determine the factors associated with the tendency to pursue a subspecialty and the level of self-perceived knowledge. The dependent variables, tendency to pursue a subspecialty (Yes = 1, No+Undecided = 0) and self-perceived knowledge (High [Detailed+General] = 1, Low [Partial+Little+None] = 0), were coded as binary categorical variables. First, all potential independent variables (gender, duration of practice, self-perceived knowledge, IC experience, opinion, appropriateness) were evaluated in univariate analyses, and variables with p < 0.25 were included in the multivariate model. The final model was created by removing non-significant variables from the model using the stepwise backward elimination method. To assess multicollinearity among the independent variables in the logistic regression models, Variance Inflation Factor (VIF) values were calculated. All VIF values were below 2.0, indicating that multicollinearity was not a significant concern in the final models. The results were reported with Odds Ratio (OR), 95% Confidence Interval (95% CI), and p-values.

To address concerns about the treatment of ordinal variables in logistic regression, we tested the linearity assumption (linearity in the logit) assumption for self-perceived knowledge levels. We compared two models: one treating self-perceived knowledge as a continuous variable (coded 0‒4) and another treating it as categorical variables using dummy coding. A Likelihood Ratio Test was conducted to assess whether the categorical model provided a significantly better fit than the continuous model. The test results indicated no statistically significant difference between the two models. This finding supports the validity of the linearity assumption, suggesting that the transitions between knowledge categories have approximately equal effects on the outcome. Therefore, treating self-perceived knowledge as a continuous variable in the logistic regression analysis is statistically justified.

The model fit was assessed with the Hosmer-Lemeshow test, and its discriminatory power was evaluated by the area under the ROC (Receiver Operating Characteristic) curve (AUC). A p > 0.05 in the Hosmer-Lemeshow test indicates that the model is compatible with the data. AUC values between 0.70‒0.80 were interpreted as acceptable, and between 0.80‒0.90 as good (reference value 0.50). A p-value of < 0.05 was considered statistically significant.

The study was approved by the Gazi Yaşargil Training and Research Hospital Clinical Research Ethics Committee (Decision n° 504, Date: 13/06/2025). The study was conducted in accordance with the principles of the Declaration of Helsinki. Electronic informed consent was obtained from all participants.

## Results

Of the 304 EM physicians participating in the study, 76.0% (n = 231) were male and 24.0% (n = 73) were female. When the duration of practice as an EM physician was examined, it was found that 61.8% (n = 188) of the participants had 0‒5 years, 15.1% (n = 46) had 6‒10 years, 18.4% (n = 56) had 11‒15 years, and 4.6% (n = 14) had 16 or more years of experience. While 80.6% (n = 245) of the participants had previous experience in patient follow-up in an IC unit, 19.4% (n = 59) did not.

When the participants' level of self-perceived knowledge about the duration and scope of IC subspecialty training was evaluated, 9.5% (n = 29) responded “I have detailed information”, 28.3% (n = 86) "I have information in general terms, “28.3% (n = 86) “I have partial information”, 24.7% (n = 75) “I have very little information”, and 9.2% (n = 28) “I have no information at all”. When the tendencies for choosing the IC subspecialty were examined, it was stated by 43.1% (n = 131) of the participants that they were considering pursuing the subspecialty, 37.2% (n = 113) were not considering it, and 19.7% (n = 60) were undecided.

When the opinions of EM physicians on whether pursuing the subspecialty would reduce the shortage of IC physicians were asked, it was expressed by 66.4% (n = 202) of the participants that this situation would reduce the shortage, 19.4% (n = 59) thought it would not, and 14.1% (n = 43) were undecided. When opinions on the appropriateness of the current IC subspecialty training were examined, it was stated by 28.6% (n = 87) of the participants that they found the training appropriate, 14.5% (n = 44) did not find it appropriate, and 56.9% (n = 173) were undecided (Table 1).

**Table 1:** Distribution of survey responses according to demographic and professional characteristics of participants.

Characteristic	n	%
Gender		
Male	231	76.0
Female	73	24.0
Years of Practice		
0‒5 years	188	61.8
6‒10 years	46	15.1
11‒15 years	56	18.4
16-years and above	14	4.6
IC Experience		
Yes	245	80.6
No	59	19.4
Self-perceived Knowledge		
Detailed knowledge	29	9.5
General knowledge	86	28.3
Partial knowledge	86	28.3
Very little knowledge	75	24.7
No knowledge	28	9.2
Tendency		
Considering	131	43.1
Not considering	113	37.2
Undecided	60	19.7
Opinion		
Will reduce	202	66.4
Will not reduce	59	19.4
Undecided	43	14.1
Appropriateness		
Yes	87	28.6
No	44	14.5
Undecided	173	56.9
Total	304	100.0

* Explanations: Years of Practice: Duration of practice as an EM physician, IC Experience: Experience in patient follow-up in IC units, Self-perceived Knowledge: Level of self-perceived knowledge about IC subspecialty training duration and scope, Tendency: Tendency to choose IC subspecialty, Opinion: Opinion on the effect of EM physicians pursuing subspecialty on IC physician shortage, Appropriateness: Opinion on the appropriateness of current IC subspecialty training. IC, Intensive Care; EM, Emergency Medicine; n, Number; %, Percentage.

A statistically significant relationship was found between the duration of practice and the tendency for the subspecialty (p < 0.001). While the tendency of physicians with 6‒10 years of experience to pursue the subspecialty was found to be higher (65.2%) compared to other groups, none of the physicians with 16 or more years of experience were considering it. A significant relationship was also found between gender and the tendency for the subspecialty (p < 0.001); the rate of indecision among female physicians (39.7%) was significantly higher than that of male physicians (13.4%).

A statistically significant relationship was observed between the level of self-perceived knowledge about the IC subspecialty and the tendency for the subspecialty (p < 0.001). While all physicians with detailed information were considering pursuing the subspecialty, it was observed that as the level of self-perceived knowledge decreased, the tendency to pursue the subspecialty also decreased. Similarly, a significant relationship was found between IC experience and the tendency for the subspecialty (p < 0.001). While 52.7% of physicians with IC experience were considering pursuing the subspecialty, only 3.4% of those without experience showed such a tendency.

The tendency for the subspecialty was found to be significantly higher among physicians who believed that the IC subspecialty would reduce the physician shortage (p < 0.001). It was also determined that the tendency for the subspecialty was significantly higher among physicians who found the current IC subspecialty training appropriate (p < 0.001) (Table 2).

**Table 2:** Association of factors with intensive care subspecialty preference tendencies.

Characteristic	Considering, n (%)	Not Considering, n (%)	Undecided, n (%)	Total (n)	p	χ², Cramer's V
Years of Practice					<0.001	64.056; 0.310
0‒5 years	73 (38.8)	57 (30.3)	58 (30.9)	188		
6‒10 years	30 (65.2)	14 (30.4)	2 (4.3)	46		
11‒15 years	28 (50.0)	28 (50.0)	0 (0.0)	56		
16-years and above	0 (0.0)	14 (100.0)	0 (0.0)	14		
Gender					<0.001	27.905; 0.292
Male	101 (43.7)	99 (42.9)	31 (13.4)	231		
Female	30 (41.1)	14 (19.2)	29 (39.7)	73		
Self-perceived Knowledge					<0.001	107.455; 0.406
Detailed knowledge	29 (100.0)	0 (0.0)	0 (0.0)	29		
General knowledge	57 (66.3)	14 (16.3)	15 (17.4)	86		
Partial knowledge	29 (33.7)	43 (50.0)	14 (16.3)	86		
Very little knowledge	16 (21.3)	42 (56.0)	17 (22.7)	75		
No knowledge	0 (0.0)	14 (50.0)	14 (50.0)	28		
IC Experience					<0.001	52.485; 0.408
Yes	129 (52.6)	70 (28.6)	46 (18.8)	245		
No	2 (3.4)	43 (72.9)	14 (23.7)	59		
Opinion					<0.001	164.624; 0.509
Yes (Will reduce)	129 (63.9)	43 (21.3)	30 (14.8)	202		
No (Will not reduce)	1 (1.7)	28 (47.5)	30 (50.8)	59		
Undecided	1 (2.3)	42 (97.7)	0 (0.0)	43		
Appropriateness					<0.001	100.156; 0.399
Yes	58 (66.7)	28 (32.2)	1 (1.1)	87		
No	0 (0.0)	14 (31.8)	30 (68.2)	44		
Undecided	73 (42.2)	71 (41.0)	29 (16.8)	173		
Total	131 (43.1)	113 (37.2)	60 (19.7)	304		

* Explanations: Years of Practice: Duration of practice as an EM physician, IC Experience: Experience in patient follow-up in IC units, Self-perceived Knowledge: Level of self-perceived knowledge about IC subspecialty training duration and scope, Opinion: Opinion on the effect of EM physicians pursuing subspecialty on IC physician shortage, Appropriateness: Opinion on the appropriateness of current IC subspecialty training. IC, Intensive Care; EM, Emergency Medicine; n, Number; %, Percentage; χ², Chi-Square test statistic; Cramer's V, Effect size coefficient measuring the strength of association between two categorical variables (0.10‒0.30: weak; 0.30‒0.50: moderate; > 0.50: strong association); p, Statistical significance level (p < 0.05 significant).

The most frequent reasons for the 131 participants considering the IC subspecialty were listed as; “expectation of better working conditions” (76.3%), “better salary and personal rights” (55.0%), and “enjoying and having a special interest in this field” (45.0%). The most frequently stated reasons by the 173 participants who were not considering or were undecided about the subspecialty were; “uncertainty and unpredictability” (42.8%), “compulsory service obligation” (42.8%), and “lack of information” (34.7%) (Table 3).

**Table 3:** Reasons for wanting and not wanting/being undecided about pursuing subspecialty.

Reasons for Wanting	n	%	Reasons for Not Wanting/Being Undecided	n	%
Expectation of better working conditions	100	76.3	Uncertainty and unpredictability	74	42.8
Better salary and fringe benefits	72	55.0	Compulsory service obligation	74	42.8
Enjoyment and special interest	59	45.0	Lack of self-perceived knowledge	60	34.7
Being complementary fields	58	44.3	Heavy workload and working conditions	59	34.1
Academic development goal	44	33.6	Belief that it will not reduce burnout	59	34.1
Abundance of job opportunities	43	32.8	Financial concerns	56	32.4
Viewing as an alternative	14	10.7	Family/personal reasons	43	24.9
Total	131	100	Mobbing concerns	33	19.1
			Length of training period	18	10.4
			Total	173	100

* Note: Participants could select multiple reasons, therefore the percentage given for each reason represents the proportion of participants who marked that reason within their own group. Reasons for wanting are calculated over n = 131, Reasons for not wanting/being undecided are calculated over n = 173. n, Number of responses; %, Percentage.

When the suggestions for improving the IC subspecialty process, which were directed to all participants, were examined, the most frequently mentioned were; “strengthening communication and collaboration” (71.1%), “increasing self-perceived knowledge” (66.8%), and “regulation of compulsory service” (66.8%) (Table 4).

**Table 4:** Suggested changes indicated by participants.

Suggestion	n	%
Strengthening communication and collaboration	216	71.1
Increasing self-perceived knowledge	203	66.8
Regulation of compulsory service	203	66.8
Improving salary and fringe benefits	187	61.5
Creating a specialized training model	161	53.0
Making entry requirements flexible	129	42.4
Reducing workload and improving working conditions	129	42.4
Shortening training duration	117	38.5

* Note: Participants could select multiple suggestions, therefore the sum of percentages exceeds 100%. Suggestions are calculated over all participants (n = 304). n, Number of responses; %, Percentage.

In the multivariate logistic regression analysis of the factors associated with the tendency to pursue a subspecialty, self-perceived knowledge and IC experience were identified as independently associated factors. Higher levels of self-perceived knowledge were significantly associated with a greater likelihood of pursuing a subspecialty. Specifically, reporting a higher category of self-perceived knowledge was associated with 3.07-times higher odds of subspecialty tendency (OR = 3.07, 95% CI 2.20‒4.29, p < 0.001). Having IC experience was associated with 6.70-times higher odds of considering a subspecialty compared to not having such experience (OR = 6.70, 95% CI 1.39‒32.27, p = 0.018). The model explains 27.2% of the variance (Pseudo *R*^2^ = 0.272). According to the Hosmer-Lemeshow test results, the model fit is good (p = 0.452). The model can discriminate the tendency for the subspecialty at a good level (AUC = 0.816, 95% CI 0.768‒0.864).

In the multivariate logistic regression analysis of the factors associated with self-perceived knowledge, IC experience was identified as the only significant associated factor. Having IC experience was associated with reporting a high level of self-perceived knowledge (OR = 44.11, 95% CI 5.99–325.08, p < 0.001). However, the extremely wide confidence interval (ranging from 5.99 to 325.08) indicates substantial model instability, likely due to quasi-complete separation in the data. This occurs because very few participants without IC experience reported high levels of self-perceived knowledge, resulting in small cell counts in one category. Consequently, while this estimate suggests a strong statistical association between IC experience and self-perceived knowledge, the magnitude of the odds ratio (44.11) should be interpreted cautiously and is presented primarily to reflect the strength of the association rather than as a precise quantitative estimate. The practical interpretation is that IC experience is a critical associated factor of self-perceived knowledge. The model explains 14.3% of the variance (Pseudo *R*^2^ = 0.143). According to the Hosmer-Lemeshow test results, the model fit is good (p = 0.891). The model can discriminate the self-perceived knowledge at an acceptable level (AUC = 0.678, 95% CI 0.618‒0.738). The variables of gender, duration of practice, opinion, and appropriateness were not found to be significant in the multivariate analysis and were removed from the final models.

To validate the assumption of linearity in the logit for self-perceived knowledge, we compared logistic regression models with self-perceived knowledge treated as continuous versus categorical. Model 1 (continuous treatment: AIC = 287.30, Log-Likelihood = −138.65) yielded a coefficient of β = 1.8594 (p < 0.001) for self-perceived knowledge. Model 2 (categorical treatment: AIC = 288.24, Log-Likelihood = −136.12) showed comparable model fit. The Likelihood Ratio Test (LR = 5.062, df = 3, p = 0.167) revealed no statistically significant difference between the models, confirming that the linearity assumption is valid for this dataset. This validation supports the use of self-perceived knowledge as a continuous variable in the subsequent multivariate analyses.

As a sensitivity analysis, a multinomial logistic regression was performed to assess the impact of combining the 'Not considering' and 'Undecided' categories. Multinomial analysis revealed important differences between these groups: greater years of practice were associated with a lower likelihood of being 'Undecided' compared to 'Not considering' (p < 0.01). Similarly, gender showed differential effects, with female physicians more likely to be 'Undecided' rather than 'Not considering' (p < 0.05). These findings indicate that the 'Undecided' group represents a distinct population with different characteristics from those definitively not considering the subspecialty. This finding supports the robustness of the primary binary analysis while acknowledging important heterogeneity within the non-considering/undecided group (Table 5).

**Table 5:** Multivariate logistic regression analysis of factors associated with subspecialty tendency and self-perceived knowledge.

Variable	β	OR	95% CI	p
Tendency ‒ Self-perceived Knowledge	1.123	3.07	2.20‒4.29	<0.001
Tendency ‒ IC Experience	1.903	6.70	1.39‒32.27	0.018
Self-perceived Knowledge ‒ IC Experience	3.787	44.11^a^	5.99‒325.08	<0.001

a This odds ratio is presented with caution; the extremely wide confidence interval indicates model instability due to quasi-complete separation in the data (see Results text for detailed interpretation). * Explanations: Tendency: Tendency to pursue IC subspecialty (Yes = 1, No+Undecided = 0), Self-perceived Knowledge: Level of self-perceived knowledge about IC subspecialty training duration and scope (High [Detailed+General] = 1, Low [Partial+Little+None] = 0), IC Experience: Experience in patient follow-up in IC units (Yes vs. No). IC, Intensive Care; β, Logistic regression coefficient, OR (Odds Ratio); OR, Odds Ratio. The ratio indicating the association between an independent variable and the odds of the dependent variable occurring (OR > 1: Higher odds, OR < 1: Lower odds, OR = 1: No association), 95% CI, Confidence interval within which the true odds ratio is expected to fall with 95% confidence; p, Statistical significance level (p < 0.05 significant).

A thematic analysis of the open-ended responses provided deeper context for the quantitative findings. 104 responses were analyzed (31 from the Concerns section and 73 from the Suggestions section). The inter-rater reliability for the qualitative coding was strong, with a Cohen's kappa of 0.84, indicating excellent agreement between the independent coders.

The predominant theme among participants' concerns was uncertainty about the future and inadequacy of current systems, encompassing fears related to post-specialization appointments, career pathways, and systemic barriers such as the compulsory service obligation. Participants expressed deep anxieties about what would happen after completing the subspecialty, the infrastructure limitations in many intensive care units, and the uncertainty of job placement.

Representative quotes from participants illustrate these concerns: “The biggest concern is the uncertainty of what happens after completing the subspecialty” (6‒10 years of experienced participant); “Compulsory service is a major barrier. The inadequate support and uncertainty about job placement make this subspecialty seem risky” (11‒15 years of experienced participant).

Correspondingly, the main themes in participants' suggestions reflected an urgent need for systematic improvements, with particular emphasis on addressing compulsory service regulations, enhancing working conditions, and strengthening training standards. Participants called for a clear and secure framework that would provide predictability and professional stability.

Representative suggestions from participants include: “Compulsory service regulations must be reformed. Without this, even better working conditions won't attract physicians” (0‒5 years of experienced participant); “Improvement in working conditions is essential. The training should be standardized, and we need clear guidelines” (6‒10 years of experienced participant).

These qualitative insights directly support and further elaborate on the motivations and barriers identified in the survey data (Table 6).

**Table 6:** Thematic analysis of open-ended responses: concerns and suggestions regarding intensive care subspecialty.

Category	Theme	Frequency (n)	Percentage (%)
Concerns	Uncertainty about future and career pathways	11	35.5
	Inadequate infrastructure and support systems	12	38.7
	Compulsory service obligation	8	25.8
	Subtotal	31	100.0
Suggestions	Reform of compulsory service regulations	18	24.7
	Improvement in working conditions	16	21.9
	Enhancement of training quality and standardization	20	27.4
	Implementation of systemic improvements	19	26
	Subtotal	73	100

* Note: n = 104 total responses analyzed (some responses contained multiple themes or could not be clearly categorized).

## Discussion

In our study, the fact that 43.1% of EM physicians are considering pursuing an IC subspecialty indicates a notable interest in this field. However, considering that our study population predominantly consists of early-career physicians, this reported tendency might be somewhat inflated, as younger physicians may be more actively seeking career development opportunities. This rate is lower than that found in the national study conducted by Aslaner et al. in Turkey in 2018, where Intensive Care was identified as the third most demanded subspecialty (67.4%).^11^ A lack of self-perceived knowledge among EM physicians regarding the IC subspecialty was also identified in the pilot study by Yıldız et al. In 2024, and this finding is consistent with the fact that 34.7% of the participants in our study stated a lack of information as a reason for not pursuing the subspecialty.^8^

In a comprehensive meta-analysis in the international literature on the factors associated with physicians' subspecialty choices, academic interest (75.29%), competencies (55.15%), and a controllable lifestyle/flexible working hours (53.00%) were listed as the three most important factors.^13^ In our study, the fact that “enjoying and having a special interest in this field” (45.0%) is among the most frequently stated reasons by those considering the subspecialty confirms that academic interest is a universal source of motivation. However, the prominence of factors such as “expectation of better working conditions” (76.3%) and “better salary and personal rights” (55.0%) in our study may stem from the unique dynamics of the healthcare system in Turkey and the dissatisfaction of physicians with their current working conditions. Additionally, the overrepresentation of early-career physicians in our sample may amplify these concerns, as they often face the most demanding shift schedules. While the effect of the income factor was determined to be 34.70% in the meta-analysis by Yang et al.,^13^ the increase of this rate to 55.0% in our study suggests that economic concerns may be more prominent among our respondents. However, direct comparison should be interpreted with caution due to differences in study populations (students vs. practicing physicians) and healthcare system contexts.

One of the most striking findings of our study is that the level of self-perceived knowledge and IC experience are strongly associated with the tendency to pursue a subspecialty. According to the logistic regression analysis results, higher self-perceived knowledge and having IC experience were strongly associated with the tendency to pursue the subspecialty (OR = 3.07 and OR = 6.70, respectively). Notably, IC experience was strongly associated with reporting a high level of self-perceived knowledge (OR = 44.11). While this odds ratio appears exceptionally high, it is important to note that this estimate is affected by quasi-complete separation, wherein very few physicians without IC experience reported high levels of self-perceived knowledge. The extremely wide confidence interval (5.99–325.08) reflects this data limitation. Nevertheless, the strong statistical significance (p < 0.001) and the consistent pattern across analyses confirm that IC experience is a key predictor of self-perceived knowledge about the IC subspecialty. A potential practical implication is that exposure to IC clinical practice substantially enhances physicians' perception of their knowledge regarding this subspecialty, regardless of the precise magnitude of the association. These findings suggest the critical role that self-perceived knowledge and experience play in career choices. These results are also consistent with the international literature. In a study conducted by Kaminski et al., it was stated that the internship experiences (41%) and mentors (21%) of medical school students were the most effective factors in changing their specialty preferences.^14^ This situation shows that in a field like IC, which requires specific and advanced self-perceived knowledge, physicians consider specializing in this area only after they feel they have attained sufficient clinical competence and experience.

It is important to acknowledge that this study measures self-perceived knowledge rather than objective knowledge, which may be subject to overconfidence bias. Furthermore, a bidirectional relationship may exist; physicians interested in pursuing the IC subspecialty may be more motivated to acquire knowledge, thus reporting higher levels of self-perceived knowledge.

Our findings align with the Theory of Planned Behavior, which posits that behavioral intention is shaped by attitudes, perceived behavioral control, and subjective norms. In our study, positive attitudes (better working conditions, salary, academic interest), perceived behavioral control (self-perceived knowledge and IC experience as associated factors), and subjective norms (desire for improved communication and cooperation) collectively associated with EM physicians' intention to pursue the IC subspecialty.

In our study, the most frequently stated reasons by participants who were not considering or were undecided about pursuing the subspecialty were “uncertainty and unpredictability” (42.8%), “compulsory service obligation” (42.8%), and “lack of information” (34.7%). These findings clearly reveal the structural problems and future anxieties faced by physicians in Turkey in their career planning. In a study by Göksu and Taşlıdere with 86 final-year medical students, the question “Would you consider emergency medicine as your first-choice specialty?” was answered negatively by 72%, and among the reasons for not choosing EM, being physically and mentally very demanding, the high number of on-call shifts, and the fear of exposure to violence were ranked highest.^15^ The study by Çetin et al. with 41 resigned EM residents supports these findings even more dramatically. In that study, violence/security concerns (63.4%), intense working environment (53.7%), and mobbing (26.8%) were identified as the most frequent reasons for resignation, with all participants stating that there were deficiencies in the training programs, and 68.2% of those who resigned continued their training in a different specialty.^16^ The fact that the most important motivation for those considering the IC subspecialty in our study was the “expectation of better working conditions” (76.3%) might possibly reflect a desire to transition away from the current emergency service conditions. This finding may suggest that some EM physicians perceive the IC subspecialty as an option not only for academic or professional satisfaction but also to achieve a career with more predictable, safer, and more humane working conditions.

The compulsory service obligation, on the other hand, appears both as a barrier for those who do not want to pursue a subspecialty and as one of the most frequently mentioned suggestions by the participants for improving the process (66.8%). As stated by Mollahaliloğlu et al., the restrictive effect of compulsory service on the lives and professional freedoms of physicians continues to be an important factor in career decisions.^17^

In our study, the fact that 66.4% of the participants see the IC subspecialty as a suitable subspecialty for EM emphasizes the close relationship and integration potential between the two disciplines. As stated by Mermiri et al., the integration of critical care services in the emergency department with models such as “resource intensivist” and “ED-ICU” can enable EM physicians to be involved in IC processes earlier and more effectively, serving as a bridge between the two disciplines.^9^

A systematic review by McDowald et al. shows that cooperation between emergency department and IC teams can be effective in reducing the mortality rates of critical patients.^18^ In this context, an increase in the number of EM-trained IC physicians will strengthen this cooperation and provide substantial improvements in patient care processes. The fact that the most frequently stated suggestions by our participants for improving the process were “strengthening communication and cooperation” (71.1%) and “increasing self-perceived knowledge” (66.8%) clearly reveals this need.

International experience, particularly in the USA since 2011, shows that EM-trained IC physicians can be successful, though employment challenges have been reported.^10^^,^^19^, ^20^, ^21^ This situation indicates that similar problems may be experienced in Turkey and is an important finding that should be taken into account in the planning of the IC subspecialty process.

Therefore, as EM physicians gain eligibility for the IC subspecialty, establishing a clear and secure legal framework for post-graduation employment, job descriptions, scope of practice, and personal rights is essential. The high level of our participants' concerns about uncertainty and unpredictability reveals the need for an urgent regulation in this regard.

The shortage of IC physicians is a serious problem worldwide.^22^, ^23^, ^24^ This situation is consistent with the findings of Pronovost et al. on the effect of intensivist physician staffing on patient outcomes.^25^ Opening the IC subspecialty to EM physicians can both contribute to closing the physician gap and create new career development opportunities for EM physicians.

## Strengths and limitations of the study

Among the strengths of this study is that it examines the tendency and self-perceived knowledge of EM physicians in Turkey to pursue an IC subspecialty using advanced statistical methods such as multivariate logistic regression analysis. Furthermore, the fact that model performance was evaluated significantly increases the reliability of the results. The thematic analysis of open-ended responses, conducted by two independent researchers, provided deeper insights into participants' concerns and suggestions, enhancing the rigor of the qualitative findings.

However, our study has several limitations. The primary limitation is the use of convenience sampling and the lack of a known denominator, which introduces selection bias. Because the survey was distributed through open networks and social media, a precise response rate, geographic, and institutional distribution could not be calculated. Notably, our sample suggests an early-career overrepresentation. Although exact national workforce demographics regarding the experience distribution of all actively working EM physicians in Turkey are not publicly available from national databases for direct comparison, we acknowledge that our sample is skewed toward younger physicians. Younger physicians may be more active online, more likely to consider pursuing a subspecialty, more affected by compulsory service obligations, and less financially stable. Additionally, the quasi-complete separation observed in the self-perceived knowledge model resulted in a very wide confidence interval for the IC experience odds ratio, which limits the precision of this particular estimate. While the association is statistically robust, the magnitude should be interpreted cautiously. Consequently, the overall interest rate in the IC subspecialty reported in this study may be inflated and should not be considered strictly generalizable to the entire national EM workforce. Due to the lack of precise and up-to-date national demographic data, we were unable to perform a stratified sensitivity analysis or re-run our regression models adjusted for experience weighting, which remains a methodological limitation of our study.

In addition, reliance on self-reported survey data may limit the objective assessment of self-perceived knowledge and attitudes, and structured response options may not fully capture the complexity of physicians' perceptions and experiences. The thematic analysis was based on a subset of participants who chose to answer the open-ended questions, which may not fully represent the views of the entire sample. Finally, the limited number of studies focusing on IC subspecialty training limits direct comparison with the existing literature.

Additionally, it is important to note that this study measures perceived needs and self-reported barriers rather than objective workforce impact or empirical economic data. The recommendations presented in this study reflect the perspectives and suggestions expressed by survey respondents, rather than evidence-based policy mandates. Therefore, these findings should be interpreted as identifying areas that may warrant further investigation and stakeholder consultation.

## Conclusion

This study suggests that there is a notable interest among EM physicians in the IC subspecialty, but this interest is met with major barriers such as lack of information, unfavorable working conditions, mobbing concerns, compulsory service obligation, and uncertainties about the future. To increase the tendency to pursue a subspecialty, it is first necessary for EM physicians to be accurately and comprehensively informed about the IC subspecialty process.

However, systemic improvements are essential: enhanced working conditions, legal protections against mobbing concerns, revised compulsory service regulations, and clear job descriptions for EM-trained IC physicians.

Future longitudinal studies can examine the changes in the career choices of EM physicians and the factors associated with these changes in more depth. In addition, studies comparing the experiences and problems faced by IC physicians from different main disciplines can contribute to the standardization of IC training and practice. Follow-up studies evaluating the employment status, professional satisfaction, and contributions to patient care outcomes of IC subspecialty graduates are of great importance.

## Ethics approval and consent to participate

The study was approved by the Gazi Yaşargil Training and Research Hospital Clinical Research Ethics Committee (Decision n° 504, Date: 13/06/2025). The study was conducted in accordance with the principles of the Declaration of Helsinki.

## Consent for publication

Not Applicable.

## Authors’ contributions

**Esat Barut:** Conceptualization, Methodology, Formal analysis, Investigation, Data curation, Resources, Writing – original draft, Writing – review & editing, Supervision. **Ramazan Giden:** Conceptualization, Methodology, Resources, Writing – review & editing, Supervision. **Kemal Barut:** Conceptualization, Methodology, Resources, Writing – review & editing, Supervision. **İbrahim Halil Yasak:** Conceptualization, Methodology, Resources, Writing – review & editing, Supervision. **Songül Araç:** Conceptualization, Methodology, Resources, Writing – review & editing, Supervision.

## Funding

Not applicable.

## Conflicts of interest

The authors declare no conflicts of interest.

## Data Availability

The datasets generated and/or analyzed during the current study are not publicly available due to participant confidentiality and ethical restrictions, but are available from the corresponding author on reasonable request, in accordance with ethical and legal requirements.
